# Development and validation of an Arabic-language Family Stability Scale for women in Saudi Arabia

**DOI:** 10.3389/fpubh.2025.1698456

**Published:** 2025-12-18

**Authors:** Mohammed A. Aljaffer, Ayedh H. Alghamdi, Ahmad H. Almadani, Norah A. Alissa, Hind A. Ababtain, Hajir S. Alhussaini, Abdulhadi A. Alhabbad, Fahad D. Alosaimi

**Affiliations:** 1 Department of Psychiatry, College of Medicine, King Saud University, Riyadh, Saudi Arabia; 2 Department of Psychiatry, King Saud University Medical City, King Saud University, Riyadh, Saudi Arabia; 3 INTHEINNER Wellbeing Center, Riyadh, Saudi Arabia; 4 Department of Psychiatry, King Abdulaziz Medical City, Riyadh, Saudi Arabia

**Keywords:** Arabic instrument, family stability, mental health, psychometric properties, Saudi women, scale validation

## Abstract

**Background:**

Family stability is a key determinant of psychological well-being; however, culturally adapted and psychometrically robust assessment tools are lacking in the Arabic context. This study aimed to develop and validate the Family Stability Scale (FSS) for Saudi women, addressing a critical measurement gap in the region.

**Methods:**

A cross-sectional psychometric validation study was conducted between January and June 2025 in Riyadh, Saudi Arabia. Women aged 18 years or older were recruited from different provinces in Saudi Arabia. The newly developed 15-item FSS, along with the Arabic versions of the Generalised Anxiety Disorder-7 (GAD-7) and Patient Health Questionnaire-9 (PHQ-9), was self-administered. Content validity was analysed via expert panel review and pilot testing. Construct validity was examined using confirmatory factor analysis. Criterion and incremental validity were assessed via correlation and hierarchical regression analyses. Reliability was evaluated through Cronbach’s alpha and split-half reliability.

**Results:**

A total of 501 women participated, with 89.8% providing complete data. Confirmatory factor analysis revealed a clear unidimensional structure, with good model fit (CFI = 0.917, TLI = 0.903, SRMR = 0.042). The FSS demonstrated excellent internal consistency (Cronbach’s *α* = 0.932) and strong split-half reliability (Spearman-Brown coefficient = 0.904). The FSS exhibited significant negative correlations with anxiety (*r* = −0.48) and depression (*r* = −0.50), indicating strong criterion validity. The scale accounted for an additional 23% of variance in anxiety symptoms and 24% in depressive symptoms beyond demographic variables, supporting its incremental validity.

**Conclusion:**

The FSS is a psychometrically sound, reliable, and valid Arabic instrument for assessing perceived family stability among Saudi women. It fills an important gap for clinical and research applications by enabling comprehensive evaluation of family functioning within the Saudi context.

## 1 Introduction

The family unit constitutes the primary social environment for human development, with the quality of its internal dynamics consistently and significantly influencing individual psychological well-being across the lifespan (1). An expanding body of international research identifies family stability is a fundamental and, crucially, modifiable protective factor against various common psychopathologies (2–4). A 10-year longitudinal study shows that mothers with high relationship stability exhibit the best mental health outcomes, whereas all other groups demonstrate at least a 3.2 times higher probability of mental health symptoms. Children of mothers from vulnerable families have an 8.2 times greater likelihood of experiencing emotional or behavioural problems compared to children of mothers with high-quality relationships (1).

The construct of family stability, however, extends beyond the simple structural integrity of the family unit. Contemporary literature has decisively shifted its focus from family composition (e.g., two-parent vs. single-parent households) (5) to the more impactful domain of perception of family functioning and coherence (6–8). This modern conceptualisation prioritises the internal emotional climate, characterised by relational quality, cohesion, and functional processes, as a more potent predictor of mental health outcomes for both adults and children (9, 10). Longitudinal data consistently show that stable, high-functioning family environments, defined by routine, open communication, and mutual support, serve as protective buffers against both mental and physical illness (11, 12). Moreover, instability within the family has been linked to broader public health concerns, including juvenile delinquency (13), academic underachievement (14), and substance abuse (15). Accordingly, public health interventions aimed at promoting mental health must look beyond structural definitions and instead focus on family functionality, a complex task necessitating the use of culturally sensitive, valid, and reliable instruments capable of assessing family functioning and stability. Based on the literature, family stability could be the perceived consistency and reliability of relational processes within the household, including predictable routines, open communication, constructive conflict resolution, and dependable mutual support. It is conceptually distinct from family structure or marital configuration (1–4, 9–12). In this study, family stability is treated as a unitary latent construct reflected by trust, cohesion, caregiving availability, effective problem solving, and participation in shared activities. Higher family stability is associated with lower emotional symptom burden and better psychosocial functioning across the lifespan.

The Kingdom of Saudi Arabia is currently undergoing unprecedented and rapid socio-cultural transformation under the framework of Saudi Vision 2030, which places women and family at the forefront of social and economic reform (16). Traditionally, Saudi families have operated within collectivistic frameworks, with extended kinship networks providing comprehensive social and emotional support. However, these families are currently experiencing significant demographic transitions. The average household size has decreased, as fertility rates dropped by 66%, from 5.78 children per woman (1980–2000) to 2.94 (2001–2021) (17). While these changes support women empowerment and economic modernisation of the nation, they introduce novel stressors that may disrupt traditional caregiving roles and family dynamics.

The increase in stressors among Saudi women is reflected in national statistics. Data from the Saudi National Mental Health Survey (SNMHS) show that 24.7% of Saudi women reported a mental disorder in the past year, with a lifetime prevalence of 35.9%, primarily involving anxiety and mood disorders. Among Saudi women, anxiety disorders were the most frequently reported 12-month and lifetime disorders, followed by mood disorders, and these conditions may significantly affect family functioning and stability (18).

As the successful and meaningful assessment of family functioning is exceptionally sensitive to cultural norms, values, and social structures (19), any measurement instrument must undergo a rigorous and comprehensive process of cross-cultural adaptation and validation. Within the Arab world, specifically in Saudi Arabia, clinicians and researchers face a measurement gap due to a deficiency of Arabic-language validated tools (20). Prominent instruments developed in Western contexts with individualistic cultures, such as the McMaster Family Assessment Device (FAD) (21), the Family Adaptability and Cohesion Evaluation Scale IV (FACES IV) (22), and the Family Environment Scale (FES) (23), may not exhibit robust psychometric performance when applied in the collectivist Saudi Arabic culture (24–26). Despite the centrality of family stability to mental health, there is no Arabic, Saudi validated instrument that operationalizes this construct with semantic and psychometric fidelity. Although family stability is a collective property, this first validation focused on women for cultural and methodological reasons. In Saudi households, women particularly mothers, typically coordinate caregiving, daily routines, communication, and support, therefore their perceptions are a sensitive proxy of the shared family climate in a collectivist context. Beginning with this key informant group enabled secure recruitment, careful linguistic refinement, and conservative testing of the factor structure. In light of these limitations, this study aimed to develop and validate a novel instrument to measure perceived family stability among Saudi women. Our primary question is whether the Arabic Family Stability Scale yields a reliable and valid measure of perceived family stability among Saudi women, evidenced by a coherent factor structure with good model fit, high internal consistency and split half reliability, and by criterion and incremental validity relative to anxiety and depression.

## 2 Methods

### 2.1 Study design and participants

**Figure 1:**
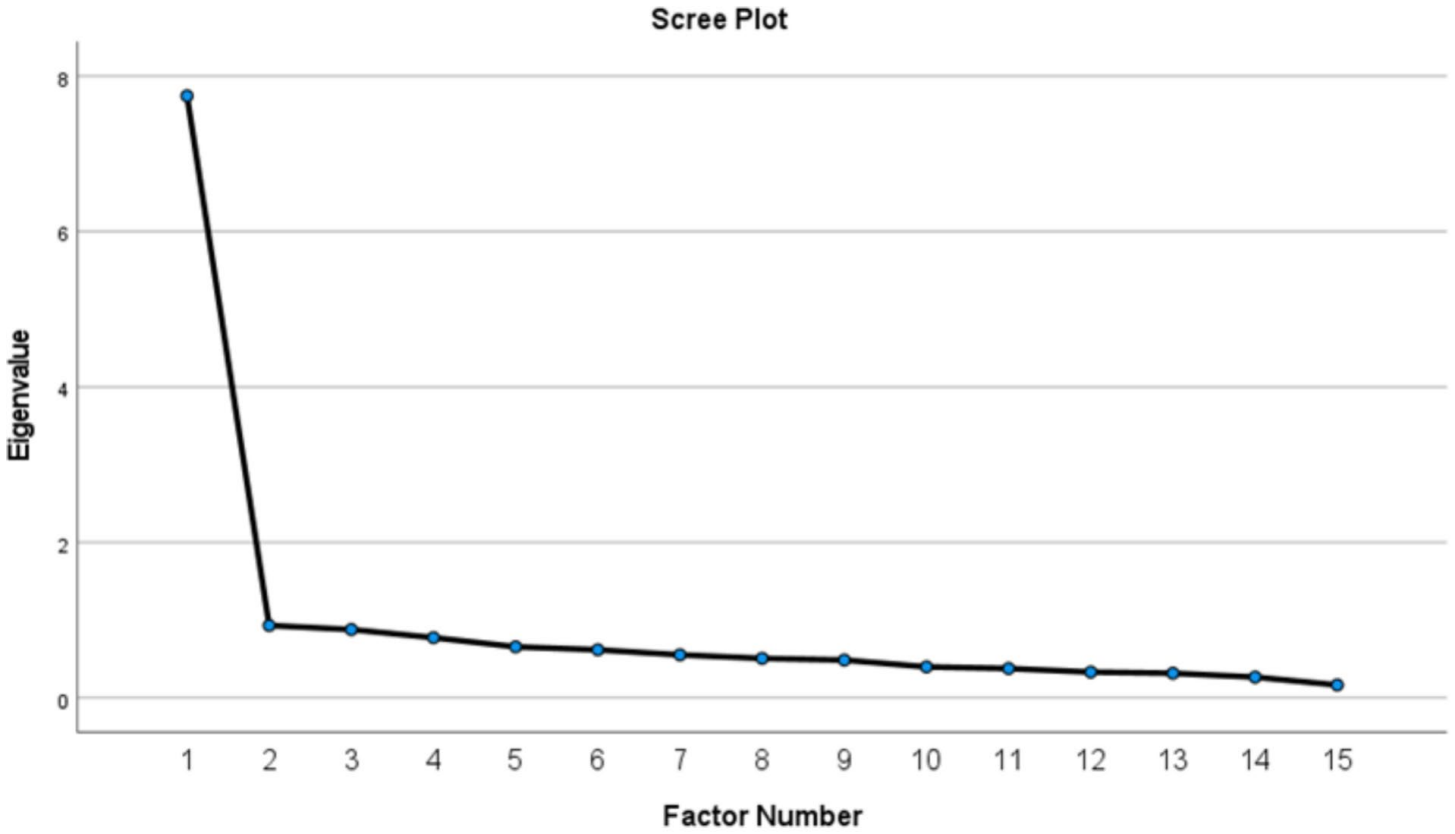
Scree plot showing eigenvalues for the Family Stability Scale.

We conducted a cross-sectional psychometric validation study. Data from each participant were collected at a single time point, aligning with the purpose of evaluating an instrument’s internal structure, reliability, and associations with external variables. The design and analysis followed established validation guidelines (27). Data were gathered from physical locations in Riyadh and extended digitally to reach a broader demographic across multiple regions across Saudi Arabia between January and June 2025. Questionnaires were self-completed in Arabic, either on paper or on secure tablets, with research staff available for clarifications. Eligible participants were Saudi women aged 18 years or older who could read Arabic and provide written consent. Individuals with cognitive or visual impairments that would prevent reliable completion of the forms were excluded to ensure respondents’ complete comprehension and response validity (Figure 1).

### 2.2 Measures

Measures included a sociodemographic form (age, marital status, household size, and monthly family income, recorded in ordered categories) and three self-report instruments. The Family Stability Scale (FSS) is a 15-item Arabic instrument developed *de novo* for this study to index perceived family stability across communication, emotional bonding, decision making, conflict and its resolution, mutual support, and shared activities. Items of FSS are rated on a 5-point Likert scale (0 = Never to 4 = Always), with higher totals indicating greater stability; the full item set appears in the Supplementary material. Emotional symptoms were assessed with the PHQ-9 and GAD-7 in Arabic, as the PHQ-9 comprises nine items scored 0–3 over the past 2 weeks (total range 0–27, using standard severity bands) and has shown good internal consistency and construct validity in Arabic-speaking samples. The GAD-7 comprises seven items scored 0–3 over the past 2 weeks (total range 0–21, with conventional cut-points) and likewise demonstrates sound reliability and validity in Arabic validations. Scoring for PHQ-9 and GAD-7 followed published guidelines (28, 29).

### 2.3 Sampling and data collection

The minimum required sample was calculated using Cochran’s formula: *n = Z^2^P(1 − P)/d^2^*, with *Z* = 1.96 (95% confidence), *p* = 0.50 (maximum variance), and *d* = 0.05 (precision). This estimation yielded a minimal sample size of 384. To meet factor-analytic criteria (≥ 10 respondents per item) and adjust for a projected 25% non-response rate, the target sample was increased to 500. A total of 501 completed questionnaires were obtained. Participants were recruited through convenience sampling, with research assistants inviting all eligible adults present during data-collection sessions until the target was reached. This approach is standard in first-stage validation studies where prevalence estimation is not the primary objective.

The main variable was the total score of the 15-item Family Stability Scale (FSS). Criterion variables were total scores from the validated Arabic versions of the Generalised Anxiety Disorder-7 (GAD-7) and Patient Health Questionnaire-9 (PHQ-9), both theoretically linked to reduced anxiety and depression (28, 29). Covariates included age (years), marital status (married/unmarried), number of household members, and monthly family income (four ordered categories).

Outcomes of interest were content validity indices, factor structure, internal consistency (Cronbach’s alpha), and criterion validity (correlation and incremental prediction of GAD-7 and PHQ-9 scores). The Arabic version of the FSS is included in the Supplementary files for other researchers. Although the original items were drafted in Arabic, an English research version was prepared to facilitate international comparison. Two bilingual translators independently created forward translations, and a third back-translated the merged version. An expert committee, including two physicians, one psychologist, and one biostatistician, reviewed all versions and resolved discrepancies to ensure semantic and conceptual equivalence.

### 2.4 Statistical analysis

Analyses were conducted using SPSS v28 and the *lavaan* package in R (version 4.3.2). After data preparation and cleaning, demographic variables were analysed descriptively using frequencies and frequencies.

To evaluate content validity, a separate panel of five domain experts rated each item’s relevance and clarity using a four-point scale. Following Zamanzadeh et al. (30), items with an item-level content validity index (I-CVI) < 0.7 were removed, those with an I-CVI between 0.70 and 0.90 were revised, and those with an I-CVI > 0.90 were retained. According to Davis (31), new instruments should have a minimum CVI of 0.80. All items exceeded these thresholds after minor revisions based on qualitative feedback. A pilot test with 30 volunteers confirmed item clarity before full data collection. The final packet included the Arabic FSS, Arabic GAD-7, and Arabic PHQ-9, which have been validated previously for Arabic-speaking samples (28, 29).

Construct validity of the FSS was evaluated using a confirmatory factor analysis (CFA) which examined whether the one-factor structure of the FSS holds when tested on an Arabic sample of mothers. The model was estimated using the *lavaan* package in *R*, including robust maximum-likelihood estimation and full-information maximum likelihood for missing data. Model fit was judged against commonly accepted cut-offs, such as Comparative Fit Index (CFI) and Tucker-Lewis Index (TLI) ≥ 0.90, Root Mean Square Error of Approximation (RMSEA) ≤ 0.08 with its 90% confidence interval, and Standardised Root Mean Square Residual (SRMR) ≤ 0.08.

The internal consistency of the FSS was assessed using Cronbach’s alpha, to determine how closely the items work together to reflect the same underlying idea. We followed a two-step approach. First, alpha was calculated on the raw responses for all 15 items. One item (“Some conflicts and disagreements arise within my family”) was phrased in the opposite direction of the others; hence, we reverse-coded this item to ensure that higher scores always indicated greater family stability. A second alpha was computed with the recoded data. Comparing the two coefficients allowed us to judge whether aligning the direction of the item improved the cohesion of the scale, as a notable improvement indicated that the item had been weakening the internal consistency in its original form. The version with the consistent scoring scheme and the higher alpha was retained for all further reliability and validity analyses. Internal consistency thresholds were interpreted as follows: ≥ 0.70 acceptable, ≥ 0.80 good, and ≥ 0.90 excellent internal consistency.

To check criterion validity, we wanted to see if individuals who felt that their family life was stable reported few emotional symptoms, which is what family-health research has conventionally found. To this end, we correlated FSS total scores with GAD-7 and PHQ-9 scores, which clinicians trust for anxiety and depression, respectively. First, we ensured that the three total scores were roughly bell-shaped – i.e., each had a skew value smaller than ±1. Having this shape allowed us to safely run ordinary Pearson correlations in R. No adjustment for multiple testing was required, as we only had two clear, theory-driven comparisons. Following common rules of thumb, correlations were interpreted as small (~0.10), moderate (~0.30), or large (≥ 0.50), with *p* < 0.05 indicating statistical significance. Finding meaningful negative correlations (higher FSS and lower GAD-7 and PHQ-9) would support the real-world or “criterion” validity of our new measure for family stability.

To test incremental criterion validity, we used a two-step hierarchical linear regression for each emotional symptom outcome. In Step 1, four demographic covariates (age, marital status, household size, and family income) were added, to account for background influences on distress. In Step 2, we added the FSS total score (15 items). This structure allowed us to determine whether family stability explained unique variance in anxiety (GAD-7) and depressive (PHQ-9) symptoms when demographic factors were controlled for. The increase in explained variance (Δ*R*^2^) was interpreted using an *α* level of 0.05 and a Δ*R*^2^ ≥ 0.02 considered practically meaningful. Regression coefficients were inspected to confirm the predicted negative direction: high family stability leads to few symptoms.

## 3 Results

A total of 501 women responded to the questionnaires; 89.8% answered almost all the FSS questions, while 10.2% provided only their demographical data (age, marital status, household size and family income) and were considered non-respondents and excluded from the final analysis. Chi-square tests found no statistically significant differences between respondents and non-respondents with regard to demographic characteristics (Table 1).

**Table 1:** Demographic characteristics of the study participants (*N* = 501).^a^

Characteristic	Category	Frequency	Percentage
Marital status	Single	44	8.8
	Married	407	81.2
	Divorced	41	8.2
	Widowed	9	1.8
Age group	18–25 years	49	9.8
	26–35 years	257	51.4
	36–45 years	137	27.4
	>45 years	57	11.4
Family size (number of household members)	≤2	113	22.6
	3–5	266	53.2
	>5	121	24.2
Family income	<10,000 SAR	314	62.7
	11,000–14,000 SAR	72	14.4
	14,000–16,000 SAR	29	5.8
	>16,000	86	17.2

a Totals may vary due to single item nonresponse; percentages are based on available cases.

### 3.1 Assessment of content validity

All five experts rated each item as either “quite relevant” or “highly relevant”, yielding I-CVI scores between 0.80 to 1.00 and a scale-level average (S-CVI average) of 0.96. No item fell below the pre-set acceptance threshold of 0.78, and after minor wording revisions. *Because the items were newly developed, a five expert panel rated relevance and clarity on a four point scale, yielding satisfactory item and scale level CVI indices, followed by a pilot with 30 volunteers to confirm comprehension. Consequently, the 15-item FSS advanced to the full study without further modifications.*

### 3.2 Assessment of construct factorial validity

The CFA indicated an adequate to good model fit for a one-factor solution. Fit indices met or approached recommended cut-offs: CFI = 0.917 and TLI = 0.903 exceeded the 0.90 benchmark, while RMSEA = 0.086 fell within the upper range of acceptability (90% CI = 0.076–0.096). The chi-square value was significant (χ^2^ = 333.9, *p* < 0.001), as expected in large samples (*N* = 450). Collectively, these statistics support the acceptability of a unidimensional structure for the FSS.

All 15 items loaded significantly onto the latent factor (*p* < 0.001), with standardised loadings between 0.45 and 0.88. The strongest indicators were Item 12 (adequate support, *λ* = 0.88), Item 11 (support in difficulty, *λ* = 0.84), and Item 10 (members care, λ = 0.78), each explaining ≥ 60% of their item variance (*R*^2^ = 0.77, 0.70, and 0.61, respectively). The reverse-coded conflict item (Item 8) had the lowest loading (λ = 0.45, *R*^2^ = 0.20) but still contributed meaningfully. Residual variances were significant yet modest for most items, indicating that the latent factor explained a substantial portion of observed variance. These findings confirm the factorial validity of the FSS as a coherent, single-dimensional construct while suggesting that Item 8 may warrant monitoring in future refinements (Tables 2, 3).

**Table 2:** Standardised factor loadings for the Family Stability Scale confirmatory factor analysis using the *lavaan* package in R.

Item	Item description	Coefficient	95% CI	*p*
1	I believe my communication with my family members is effective.	0.63	[0.56, 0.70]	<0.001
2	My family members comfortably discuss beliefs and values.	0.61	[0.53, 0.68]	<0.001
3	I feel that my family members express their feelings and love for each other.	0.68	[0.61, 0.74]	<0.001
4	I feel a strong emotional bond with my family members.	0.68	[0.62, 0.75]	<0.001
5	I feel that my family members react well to my feelings, such as anger and sadness.	0.76	[0.71, 0.81]	<0.001
6	I feel a sense of trust and credibility within my family.	0.75	[0.70, 0.81]	<0.001
7	I see or sense a great deal of compromise in decision-making within my family.	0.55	[0.46, 0.63]	<0.001
8	Some conflicts and disagreements arise within my family (reverse-coded)	0.45	[0.36, 0.53]	<0.001
9	These emerging conflicts and disagreements within my family are resolved effectively.	0.70	[0.63, 0.76]	<0.001
10	I feel that my family members care for each other.	0.78	[0.74, 0.83]	<0.001
11	I feel that my family members are there for me when I face difficulties.	0.84	[0.80, 0.87]	<0.001
12	I feel that I receive adequate support from my family members.	0.88	[0.85, 0.91]	<0.001
13	I feel that my family members support and accept my desire to pursue new paths or projects.	0.70	[0.65, 0.76]	<0.001
14	My family members participate in celebrations together, such as celebrating Eid al-Fitr.	0.61	[0.54, 0.68]	<0.001
15	My family members participate in recreational activities together.	0.69	[0.63, 0.74]	<0.001

**Table 3:** Goodness-of-fit indices for the one-factor confirmatory factor analysis model of the Family Stability Scale.

Fit index	Obtained value	Recommended cut-off points^a^	Interpretation
χ^2^ (df = 90)	333.87	–	Significant (expected with large N)
Comparative Fit Index (CFI)	0.917	≥0.90 (good)	Good fit
Tucker–Lewis Index (TLI)	0.903	≥0.90 (good)	Good fit
Root Mean Square Error of Approximation (RMSEA)	0.086	≤0.08 (reasonable)	Upper end of reasonable fit
Standardised Root Mean Square Residual (SRMR)	0.042	≤0.08 (good)	Good fit

a Cut-off guidelines follow Hu and Bentler (49).

In terms of criterion validity, the FSS exhibited the expected inverse relationship with emotional symptom burden. Pearson analysis revealed a moderate-to-large negative correlation with anxiety (FSS × GAD-7, correlation coefficient (*r*) = −0.48, *p* < 0.001) and a slightly stronger negative correlation with depressive symptoms (FSS × PHQ-9, correlation coefficient (*r*) = −0.50, *p* < 0.001). Participants who perceived their family life as stable reported notably low levels of anxiety and depressive symptoms.

These coefficients exceed the conventional threshold of 0.30 for a moderate effect and approach the 0.50 benchmark often deemed large, providing clear evidence that the new scale behaves in line with theory and clinical observation. As both correlations were strong and highly significant in the expected direction, the results support the criterion validity of the FSS: it effectively distinguishes individuals with higher perceived family cohesion from those experiencing elevated emotional distress.

Adding the FSS total score significantly enhanced the prediction of both mental health outcomes. For anxiety, the explained variance of the model increased by approximately 23% (change in *F* = 123, *p* < 0.001) after the stability score was introduced, and the coefficient for family stability was negative and highly significant (*β* = −0.23, *p* < 0.001). Similarly, for depressive symptoms, the FSS accounted for an additional 24% of explained variance (change in *F* = 127, *p* < 0.001), with a similarly strong negative coefficient (*β* = −0.29, *p* < 0.001). In both models, demographic variables had limited effects, while family stability remained the most influential predictor. These results confirm the incremental criterion validity of the FSS, indicating that it offers unique and substantial exploratory power beyond basic demographic factors.

### 3.3 Assessment of the internal consistency of the Family Stability Scale using Cronbach’s alpha

The FSS exhibited high internal consistency across the 15 items (Cronbach’s *α* = 0.91). It indicates that the items collectively measure a cohesive construct of perceived family stability. For the majority of the items, the corrected item-total correlations ranged from 0.55 to 0.83, exceeding the widely accepted threshold of 0.30. These values demonstrate that individual items are substantially correlated with the overall scale and contribute positively to its internal coherence. The highest corrected correlation was observed for Item 12 (“I feel that I receive adequate support from my family members”). Items 10 and 11, namely “Family members care for each other” and “Family availability during difficulties”, also showed strong correlations, highlighting the centrality of emotional and instrumental support in defining family stability.

However, Item 8 (“Some conflicts and disagreements arise within my family”) exhibited a negative corrected item-total correlation (−0.43) and the highest “Cronbach’s Alpha if Item Deleted” value (0.93), suggesting that it was misaligned with the overall scale. While this misalignment may reflect its focus on a potentially protective or normative aspect of family dynamics, such as the occurrence of conflict, its statistical inconsistency indicated a conceptual divergence from the scale’s general direction. Nonetheless, the item was retained in its original form to preserve content validity and to reflect a broader spectrum of family experiences, including relational tension.

After reverse-coding Item 8, the internal consistency of the FSS improved notably, with Cronbach’s alpha increasing from 0.910 to 0.932. This adjustment aligned the item directionally with the rest of the scale, resulting in a positive corrected item-total correlation of 0.431, compared to −0.43 prior to reversal. Although this correlation remained relatively low, it became a constructive contributor to the scale’s internal structure. Additionally, the “Cronbach’s Alpha if Item Deleted” value for Item 8 remained unchanged, indicating that it no longer inflated internal inconsistency. The analysis further that eliminating any item did not improve overall reliability, supporting the conclusion that all items contribute meaningfully to the scale. These findings suggest that Item 8 was conceptually sound but required directional adjustment to match the positive orientation of the scale, underscoring the importance of consistent coding when including negatively framed items that address normative family processes such as conflict (Table 4).

**Table 4:** Comparison of item-level reliability metrics before and after reverse-coding item 8 in the Family Stability Scale.

Item No.	Item description (shortened)	Corrected item-total correlation (before)	Corrected item-total correlation (AFTER)	Cronbach’s alpha if item deleted (before)	Cronbach’s alpha if item deleted (after)
1	I believe my communication with my family members is effective.	0.626	0.635	0.904	0.928
2	My family members comfortably discuss beliefs and values.	0.593	0.595	0.905	0.929
3	I feel that my family members express their feelings and love for each other.	0.656	0.663	0.902	0.927
4	I feel a strong emotional bond with my family members.	0.660	0.668	0.902	0.927
5	I feel that my family members react well to my feelings, such as anger and sadness.	0.720	0.730	0.900	0.925
6	I feel a sense of trust and credibility within my family.	0.724	0.732	0.900	0.925
7	I see or sense a great deal of compromise in decision-making within my family.	0.547	0.530	0.906	0.931
8	Some conflicts and disagreements arise within my family.	−0.431	0.431	0.933	0.933
9	These emerging conflicts and disagreements within my family are resolved effectively.	0.660	0.685	0.902	0.926
10	I feel that my family members care for each other.	0.744	0.756	0.900	0.925
11	I feel that my family members are there for me when I face difficulties.	0.783	0.785	0.897	0.923
12	I feel that I receive adequate support from my family members.	0.827	0.832	0.896	0.922
13	I feel that my family members support and accept my desire to pursue new paths or projects.	0.660	0.658	0.902	0.927
14	My family members participate in celebrations together, such as celebrating Eid al-Fitr.	0.586	0.585	0.905	0.929
15	My family members participate in recreational activities together.	0.677	0.671	0.902	0.927
	Cronbach’s alpha (full scale)	0.910	0.932		

The split-half reliability analysis further supported these findings, highlighting the internal consistency of the FSS. The strong inter-half correlation (*r* = 0.825) and the values of Spearman-Brown and Guttman coefficients at 0.904 indicated excellent reliability even when accounting for unequal item distribution. These findings align with the high Cronbach’s alpha value (0.932), reflecting the scale’s robustness and internal coherence across all 15 items. For GAD-7 and PHQ-9 Arabic instruments, we found excellent internal consistency (GAD-7: Cronbach’s *α* = 0.900, standardised α = 0.902; PHQ-9: Cronbach’s α = 0.903, standardised α = 0.903). Split-half analyses also indicated high reliability (inter-half *r* = 0.887 and 0.900 for GAD-7 and PHQ-9, respectively), with corresponding Spearman–Brown coefficients of 0.940 and 0.947, respectively.

## 4 Discussion

The primary objective of this study was to develop and undertake a rigorous psychometric validation of the FSS, a new 15-item instrument designed to assess perceived family stability among Arabic-speaking women in Saudi Arabia. The clinical significance of family functioning in mental health (1, 3–5, 10), alongside the Kingdom’s unique socio-cultural transitions (16, 18, 19, 32) and the inadequacy of existing Western-developed measurement tools (24–26), underscore the necessity of this study. The absence of a culturally and psychometrically robust scale further highlights its importance. While we argue that direct transplantation of Western instruments may misfit collectivist settings, our design does not reintroduce individualism. This study represents the first phase of a staged validation strategy. We began with women as key informants because they typically coordinate caregiving, daily routines, and emotional support in Saudi households, which yields a high information signal for the shared family climate. The Family Stability Scale is worded in a gender neutral and role agnostic manner, and the construct is defined at the household level.

Existing translated tools in Arabic have serious limitations. For example, the Arabic version of the Family Assessment Measure III (FAM III) (25), validated in a Saudi sample, showed a statistically poor model fit to the collected data. Acceptable model fit was achieved only after a drastic and highly problematic statistical intervention, which required deleting half of the scale’s original items due to poor factor loadings. The researchers concluded that these limitations likely reflect cultural differences in how family functioning is conceptualised (25).

Similarly, a Saudi validation of the FACES IV required significant statistical modifications to the theoretical model in order to achieve acceptable fit with the data (26). This finding supports a broader body of cross-cultural evidence suggesting that Western theoretical constructs do not always translate effectively across cultures (33). Furthermore, although the Family APGAR, a brief 5-item screening tool, has an Arabic version that has been found feasible for rapid clinical screening, it lacks the psychometric depth required for comprehensive assessment of family functioning or stability (24).

In this validation study, the FSS yielded strong evidence of reliability and validity. The scale demonstrated excellent internal consistency (Cronbach’s *α* = 0.932) and strong split-half reliability (Spearman-Brown coefficient = 0.904). Construct validity was robustly supported through CFA, showing good model fit (CFI = 0.917, TLI = 0.903, SRMR = 0.042). Furthermore, criterion validity was substantiated through moderate-to-strong negative correlations with anxiety (*r* = − 0.48) and depression (*r* = −0.50). Incremental validity was demonstrated by the FSS accounting for an additional 2–24% of the variance in mental health symptoms beyond demographic variables. Collectively, these findings affirm the FSS as a psychometrically sound instrument for assessing perceived family stability among Saudi women.

### 4.1 Comparison with validated scales in the Arab context

The psychometric properties of the FSS compare favourably with those of other family functioning scales, particularly within the Arab or Saudi context. The internal consistency of the FSS (Cronbach’s *α* = 0.932) exceeded the reliability estimates reported for the Arabic version of the FAM III in Saudi Arabia, where alpha coefficients ranged from 0.68 to 0.72, indicating only moderate reliability (26). The divergence in construct validity was even more pronounced. The FSS yielded a clear, interpretable unidimensional factor structure. In contrast, the validation of the seven-factor FAM III in a Saudi sample reported poor initial model fit, requiring the deletion of half of the original items and the inclusion of numerous correlated errors to achieve an acceptable structure. The authors of that study explicitly noted that these statistical issues likely reflected cultural differences (26), thereby supporting the culturally-grounded approach adopted for the FSS.

Comparable challenges have been identified with the FACES IV in the region (13). A Saudi validation of the FACES IV also required substantial statistical adjustments to achieve acceptable model fit, with the study highlighting “particularities with the rigid and enmeshed dimensions”. This finding is critical, as it suggests a conceptual misalignment between Western theoretical constructs and the Saudi cultural context. The unidimensionality of the FSS implies that, for Saudi women, various domains of family life, such as communication, support, and conflict resolution, are not experienced as distinct but rather as integrated elements of a single, holistic construct of family stability. This finding contrasts with the multidimensional frameworks employed in Western models and suggests that the FSS is more conceptually aligned with the collectivistic cultural experiences of family life.

Other scales validated in Arabic-speaking populations further contextualise the FSS’s contribution. For instance, the Perceived Collective Family Efficacy Scale, validated in an Algerian sample, also produced a unidimensional structure with good reliability (Cronbach’s *α* = 0.898), suggesting that single-factor models may be well suited to Arab cultures (34). While the Arabic version of the Family APGAR has been used across Arab countries (24), it remains a brief 5-item screening tool. In contrast, the 15-item FSS offers greater psychometric depth, making it more suitable for comprehensive research and nuanced clinical evaluation. An Arabic version of the McMaster FAD has also been utilised in Saudi Arabia and other Arab countries (35, 36). However, comprehensive, published full-scale validation data in this specific population remain limited.

The conceptual structure of the FSS can be related to major international models of family functioning. The most prominent of these is the Circumplex Model, which underpins the FACES instruments (37). This model defines healthy family functioning through cohesion (emotional bonding) and adaptability (flexibility), positing that extremes in either dimension (e.g., enmeshed, rigid, chaotic) may be dysfunctional (38). It assumes a linear relationship between these dimensions and psychological well-being. The FSS, in contrast, demonstrated a robust unidimensional structure, indicating that, in the Saudi context, these constructs are perceived not as separate but as interwoven. The FSS items – support, communication, and emotional bond – appear so closely integrated that they are interpreted as a single underlying trait of stability.

Another key international instrument, the McMaster FAD, is based on the McMaster Model of Family Functioning (39), which evaluates six domains: problem solving, communication, roles, affective responsiveness, affective involvement, and behavioural control, along with a general functioning scale. While the FAD allows for detailed, multi-faceted assessment, the FSS’s strong unidimensionality and high internal consistency (*α* = 0.932) suggest that these different domains may be experienced as a unified construct by Saudi women. The FSS’s single-factor structure may, therefore, be more reflective of a holistic cultural view of family functioning, wherein the quality of one domain cannot be separated from the others.

The FES provides another point of comparison (40). This 90-item instrument assesses 10 subscales across three dimensions: relationship, personal growth, and system maintenance. However, concerns have been raised regarding the FES’s psychometric integrity, with reports of low internal consistency for certain subscales and inconsistent support for its theoretical structure (41–43). In this respect, the FSS, with its demonstrated high reliability and clear, replicable factor structure, offers a more concise and psychometrically robust alternative for assessing perceptions of family functioning in the Saudi context.

The FSS can also be examined in relation to instruments based on Walsh’s Family Resilience Framework, such as the Family Resilience Assessment Scale (FRAS) (44). Walsh’s model is multidimensional, encompassing family belief systems, organisational patterns, and communication processes. Validation studies of the FRAS have yielded varying factor structures across cultures, again highlighting the challenge of developing universally applicable multidimensional measures (44–46). While resilience is indeed multifaceted, the FSS specifically targets the perceived state of *stability* – defined by consistent and predictable routines and relationships – which itself constitutes a core element of resilience. The FSS thus captures a foundational component that enables families to effectively manage adversities.

Finally, the criterion validity of the FSS aligns well with the international literature. The observed moderate negative correlations with depression (*r* = −0.50) and anxiety (*r* = −0.48) mirror results from numerous studies linking family dysfunction with mental disorders. For example, a review examining family functioning in the context of paediatric pain reported correlations with depression as high as *r* = 0.52 and with anxiety as high as *r* = 0.43 (47). The magnitude of the associations found in this study positions the FSS as a clinically relevant instrument for evaluating family functioning.

The validation of the FSS carries significant practical implications for clinical practise in Saudi Arabia. The Kingdom is undergoing substantial expansion in its mental health services, particularly within primary healthcare (PHC). By 2022, approximately 75% of PHC centres were delivering mental health services, reflecting a national commitment to accessibility (48). As a brief, reliable, and easily administered tool, the FSS is well suited to this environment. PHC physicians and family medicine practitioners could incorporate the FSS into routine screening to identify women and families with low perceived stability, who may be at elevated risk for depression or anxiety. Early detection would support timely psychoeducation, counselling, or referral to more specialised family therapy services, in line with preventative care priorities. Given the high lifetime prevalence of mental disorders among Saudi women, a measure that facilitates discussion of the family context is particularly valuable. In research, the FSS offers a tool for longitudinal studies examining how perceived family stability evolves in response to social change and which aspects are most protective. It may also serve to evaluate the impact of family-focused interventions designed to enhance women’s well-being and mental health.

A major strength of this study lies in its rigorous, two-stage validation process, adhering to established standards for instrument development. The large sample size (*N* = 501) afforded ample statistical power, well exceeding the common threshold of 10 participants per item. Furthermore, the assessment of criterion validity against well-established, validated scales for depression (PHQ-9) and anxiety (GAD-7), along with the demonstration of incremental validity via hierarchical regression, provide strong evidence that the FSS measures a clinically meaningful construct significantly associated with real-world mental health outcomes.

### 4.2 Limitations of the study

Despite its strengths, this study has several limitations that should be considered while interpreting the results. The study employed convenience sampling. Furthermore, the sample comprised exclusively women. While it was an intentional focus on a key demographic, the findings cannot be generalised to men, whose perceptions and experiences of family stability may differ. Future research aims to validate the FSS with more diverse, nationally representative samples including men. *Because this first validation was conducted in women, generalizability to men remains to be tested, and future work should include father and children participants and family-level reports, with multigroup confirmatory factor analysis and differential item functioning*. Future studies should also examine test–retest reliability and predictive validity over time. Finally, all measures relied on self-report, which may be influenced by social desirability bias and other response biases. Although self-perception is the target construct, incorporating observational data or reports from other family members in future studies can yield a more comprehensive evaluation of family functioning.

## 5 Conclusion

This validation study offers robust psychometric evidence supporting the FSS as a reliable and valid measure of perceived family stability among women in Saudi Arabia. The scale demonstrated excellent internal consistency and reliability, with high coherence across items and strong split-half reliability. Factorial validity was clearly established through confirmatory factor analysis, which consistently supported a clear, cohesive unidimensional structure. Moreover, the scale exhibited substantial criterion and incremental validity, demonstrated by significant inverse associations with anxiety and depression measures as well as its capacity to explain additional variance in mental health symptoms beyond demographic variables alone. Collectively, these findings affirm the FSS as a psychometrically sound instrument suitable for clinical and research applications, effectively capturing the multifaceted aspects of family stability within the Saudi cultural context.

## Data Availability

The raw data supporting the conclusions of this article will be made available by the authors, without undue reservation.
