# Supplementary material for: Development and validation of an Arabic-language Family Stability Scale for women in Saudi Arabia
Source: Front Public Health. 2025 Dec 18;13:1698456. doi: 10.3389/fpubh.2025.1698456 (PMC12756151; doi:10.3389/fpubh.2025.1698456)

Supplementary Material

Development and Validation of an Arabic Family Stability Scale among Women in Saudi Arabia

# Supplementary Table

The original scale : Validated Family Stability Scale (15-item) in Arabic

| **قياس الاستقرار الأسري** | | | | | |  |
| --- | --- | --- | --- | --- | --- | --- |
| ***أبداً*** | ***نادرًا*** | ***أحيانا*** | ***معظم الأوقات*** | ***دائمًا*** | ***الفقرة*** | ***البعد*** |
|  |  |  |  |  | **أعتقد أن تواصلي مع أفراد اسرتي فعال .** | **التواصل** |
|  |  |  |  |  | **أفراد عائلتي يجرون مناقشات بخصوص المعتقدات والقيم بأريحية .** |  |
|  |  |  |  |  | **أشعر أن أفراد اسرتي يعبرون عن مشاعرهم وحبهم تجاه بعضهم البعض .** | **الرابطة العاطفية** |
|  |  |  |  |  | **أشعر بارتباط عاطفي قوي بأفراد اسرتي .** |  |
|  |  |  |  |  | **أشعر أن أفراد أسرتي يتفاعلون بشكل جيد مع مشاعري، كالغضب والحزن .** |  |
|  |  |  |  |  | **أشعر بالثقة والمصداقية داخل أسرتي .** | **اتخاذ القرارات** |
|  |  |  |  |  | **أرى أو استشعر قدرًا كبيرًا من التنازلات فيما يتعلق في اتخاذ القرارات داخل أسرتي .** |  |
|  |  |  |  |  | **هناك بعض النزاعات و الخلافات التي تنشأ داخل اسرتي .** | **الخلافات** |
|  |  |  |  |  | **يتم حل هذه النزاعات والخلافات الناشئة داخل أسرتي بشكل فعال .** |  |
|  |  |  |  |  | **أشعر أن أفراد أسرتي يهتمون برعاية بعضهم البعض .** | **الدعم** |
|  |  |  |  |  | **أشعر أن أفراد أسرتي متواجدين بجانبي عندما أواجه الصعوبات .** |  |
|  |  |  |  |  | **أشعر أنني أتلقى الدعم الكافي من أفراد أسرتي .** |  |
|  |  |  |  |  | **أشعر أن أفراد أسرتي يدعمون ويتقبلون رغبتي في التوجه نحو مسارات أو مشاريع جديدة .** |  |
|  |  |  |  |  | **يشارك أفراد أسرتي في الاحتفالات معًا، كالاحتفال بعيد الفطر .** | **الأنشطة المشتركة** |
|  |  |  |  |  | **يشارك أفراد أسرتي معا في الأنشطة الترفيهية .** |  |


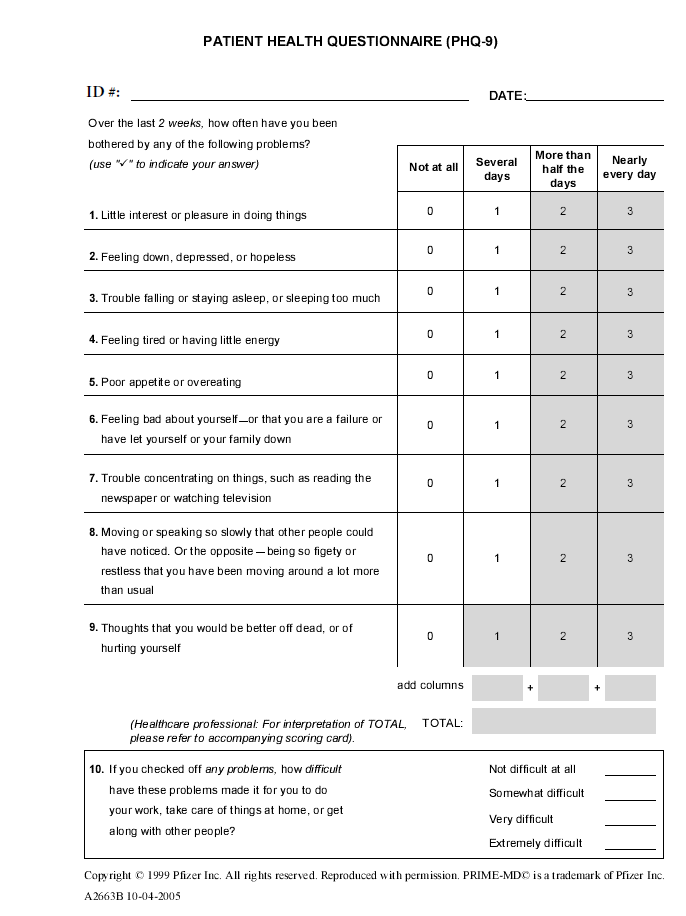


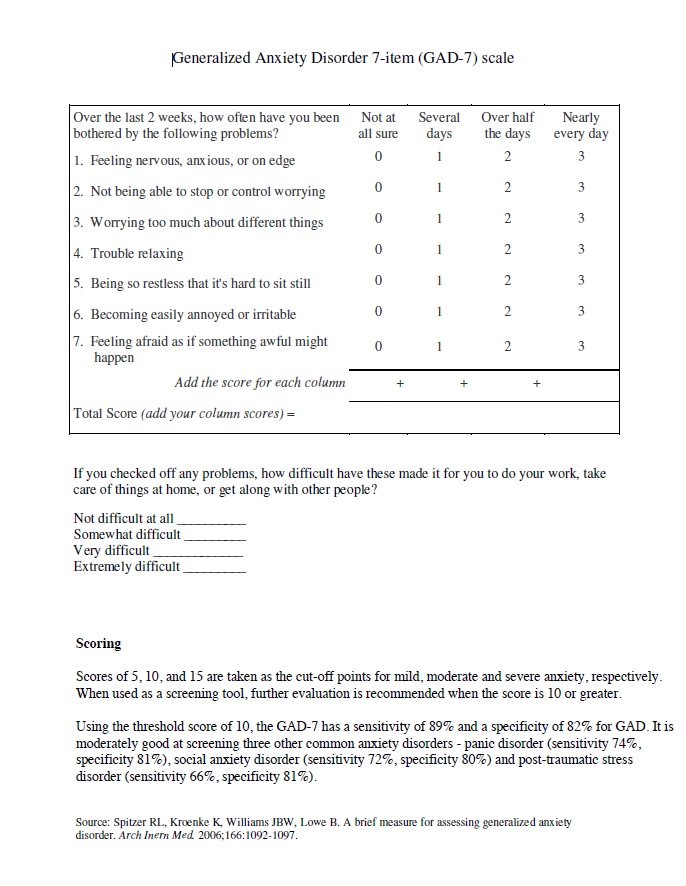

Supplement: Supplementary file 1 [file Table_1.DOCX]
